# Diversifying of Chemical Structure of Native *Monascus* Pigments

**DOI:** 10.3389/fmicb.2018.03143

**Published:** 2018-12-21

**Authors:** Lujie Liu, Jixing Zhao, Yaolin Huang, Qiao Xin, Zhilong Wang

**Affiliations:** ^1^State Key Laboratory of Microbial Metabolism, Engineering Research Center of Cell and Therapeutic Antibody, Ministry of Education, School of Pharmacy, Shanghai Jiao Tong University, Shanghai, China; ^2^Shandong Zhonghui Biotechnology Co., Ltd., Binzhou, China

**Keywords:** Red Yeast Rice, *Monascus* pigments, non-*Monascus* species, chemical modification, enzyme inhibitor

## Abstract

Red Yeast Rice, produced by solid state fermentation of *Monascus* species on rice, is a traditional food additive and traditional Chinese medicine. With the introduction of modern microbiology and biotechnology to the traditional edible filamentous fungi *Monascus* species, it has been revealed that the production of red colorant by fermentation of *Monascus* species involves the biosynthesis of orange *Monascus* pigments and further chemical modification of orange *Monascus* pigments into the corresponding derivates with various amine residues. Further study indicates that non-*Monascus* species also produce *Monascus* pigments as well as *Monascus*-like pigments. Based on the chemical modification of orange *Monascus* pigments, the diversification of native *Monascus* pigments, including commercial food additives of Red *Monascus* Pigments^®^ and Yellow *Monascus* Pigments^®^ in Chinese market, was reviewed. Furthermore, *Monascus* pigments as well as their derivates as enzyme inhibitors for anti-obesity, hyperlipidemia, and hyperglycemia was also summarized.

## Introduction

Filamentous fungi, known as a prolific producer of secondary metabolites, are an important resource for discovering small molecules of pharmaceutical (such as penicillin, lovastatin, cyclosporine, etc.) as well as food colorant additives (Dufossé et al., 2014). One of the famous examples is the production of Red Yeast Rice (a traditional Chinese food colorant and medicine) by *Monascus* species (Ma et al., 2000). *Monascus* species were first screened in Red Yeast Rice and characterized in 1884 (van Tieghem, 1884). After then, modern microbiology and biotechnology are introduced to the traditional edible filamentous fungi. Many secondary metabolites of *Monascus* species are isolated and identified, such as monacolin K (Endo, 1980), citrinin (Blanc et al., 1995), and *Monascus* pigments. At the same time, the negative and positive bioactivity of the secondary metabolites are also recognized. There are many comprehensive reviews about the molecular biology of *Monascus*, the secondary metabolites as well as their metabolite bioactivity (Juzlova et al., 1996; Feng et al., 2012; Chen et al., 2015).

**FIGURE 1:**
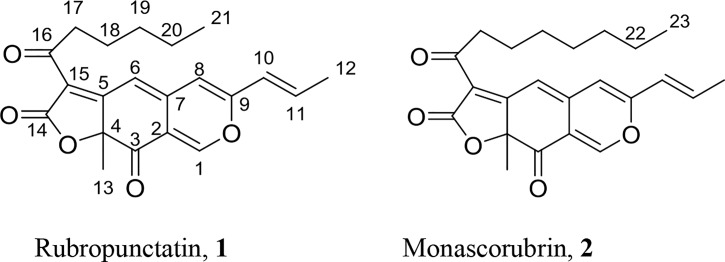
Chemical structure of orange *Monascus* pigments.

Orange *Monascus* pigments, including rubropunctatin (**1**) and monascorubrin (**2**) with a classic azaphilone structure (Figure 1), are key color components of *Monascus* fermentation. In the present work, we focus on the structure diversity of orange *Monascus* pigment derivates as well as their corresponding characters and bioactivities. First, progress on the biosynthetic pathway of orange *Monascus* pigments was updated. Then, production of orange *Monascus* pigments as well as *Monascus*-like pigments by non-*Monascus* species was introduced. And then, the diversification of *Monascus* pigment structure, including commercial food additives of Red *Monascus* Pigments^®^ and Yellow *Monascus* Pigments^®^ in Chinese market, by chemical modification of orange *Monascus* pigments was reviewed. Finally, *Monascus* pigments as well as their derivates as enzyme inhibitors for anti-obesity, hyperlipidemia, and hyperglycemia was also summarized.

## Biosynthesis of *Monascus* Pigments

*Monascus* pigments, such as rubropunctatin (**1**) and monascorubrin (**2**), are chemicals by the esterification of azaphilone with β-ketoacid. The biosynthesis of β-ketoacid is catalyzed by fatty acid synthase (FAS) while azaphilone is catalyzed by polyketide synthase (PKS). Both polyketides and fatty acids are architected by iteratively decarboxylative Claisen thioester condensations of an activated acyl starter unit with malonyl-CoA-derived extender units (Smith and Tsai, 2007). The difference is that FAS typically catalyzes a full reductive chain after each elongation while PKS catalyzes an optional reductive cycle that can be partly or fully omitted before the next round of elongation (Hertweck, 2009). Nuclear magnetic resonance analysis after feeding with ^13^C-labeled acetate during submerged cultures is applied to investigate the biosynthetic pathway of *Monascus* pigments. Results indicate that fatty acid biosynthesis and polyketide biosynthesis shares acetate and propionate as the simplest biosynthetic building blocks (Hajjaj et al., 2000). Furthermore, it is also confirmed that tetraketide is the precursor of *Monascus* pigments in the biosynthetic pathway (Hajjaj et al., 1999). Recently, the biosynthetic pathway of *Monascu*s pigments becomes more and more clear with the progress of modern molecular biology (Balakrishnan et al., 2013; Chen et al., 2017).

### Biosynthetic Pathway of *Monascus* Pigments

**FIGURE 2:**
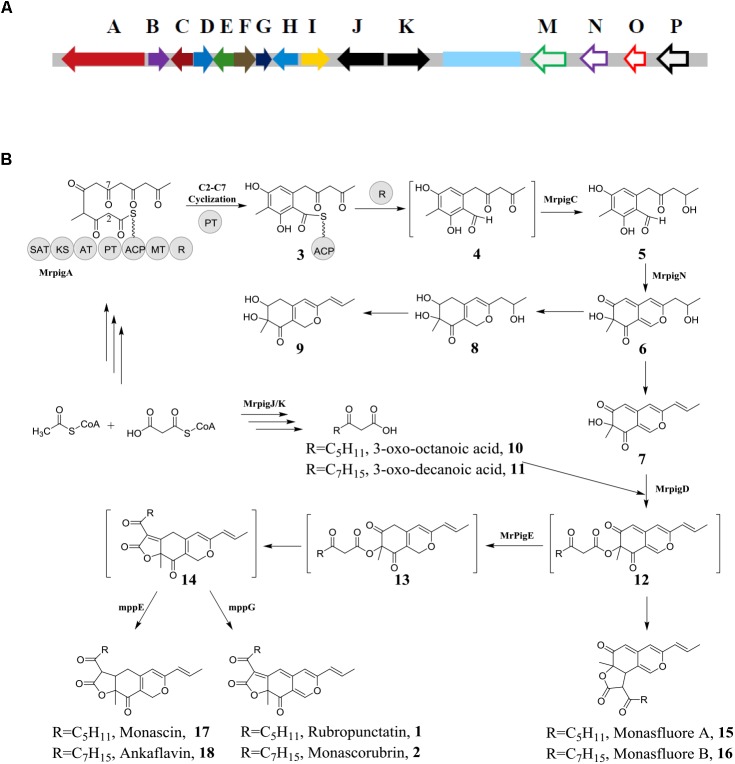
Biosynthesis of orange *Monascus* pigments. **(A)** Conserved genes involving in biosynthesis of *Monascu*s pigments; **(B)** biosynthetic pathway of *Monascu*s pigments.

Gene cultures for biosynthesis of *Monascus* pigments had been studied extensively by two groups, i.e., Chen’s group of Huazhong Agricultural University (China) (Chen et al., 2017) and Kwon’s group of Myongji University (South Korea) (Balakrishnan et al., 2013). Based on the gene cultures from various *Monascus* species, some highly conserved and nearly identical genes in all *Monascus* species are summarized (Figure 2A). Those genes are divided into two regions, i.e., region I indicating as filled arrows and region II as open arrows, which encode the core enzymes for *Monascus* pigment biosynthesis. Between region I and region II, there are some genes for special *Monascus* species, which is shown as light blue box in Figure 2A. For examples, there is an ankyrin repeat protein-encoding gene in *M. ruber* M7 and two *M. purpureus* strains. *M. rube*r NRRL 1597 and *M. pilosus* contain six genes related to transport and signal transduction (Chen et al., 2017).

Among the gene clusters of region I, Mrpig A gene, corresponding to MpPKS5 gene of *Monascus purpureus* (Balakrishnan et al., 2013), encodes PKS and catalyzes biosynthesis of the key aromatic ring intermediate (**4**) (Figure 2B). Similarly, Mrpig N gene in region II, corresponding to mpp 7 gene of *Monascus purpureus* (Balakrishnan et al., 2014a), encodes a FAD-dependent monooxygenase and catalyzes the hydroxylation reaction at the C-4 carbon of compound **5**. Thus, the key azaphilone structure chemical **6** can be biosynthesized. Many filamentous fungi are sources of secondary metabolites with bicyclic azaphilone structures. Similar gene clusters are also identified, such as *Aspergillus niger* (Zabala et al., 2012). Different from other filamentous fungi, there is Mrpig E gene in region II of *Monascus* gene culters, corresponding to mpp C gene of *Monascus purpureus* (Balakrishnan et al., 2014b), encodes NAD(P)H-dependent oxidoreductase and catalyzes the reduction of compound **12** into **13**. The chemical **13** is the key to formation of the tricyclic ring of *Monascus* pigments. The pathway for biosynthesis of *Monascus* pigments is detailed below.

### Biosynthesis of Azaphilone Structure

Fungal PKSs can be classified into three groups according to the function and phylogeny, i.e., non-reducing PKSs, partial-reducing PKSs, and highly reducing PKSs (Chooi and Tang, 2012). The biosynthesis of *Monascus* pigments starts with the assembly of a hexaketide backbone catalyzed by the non-reducing PKS encoded by Mrpig A gene. As shown in Figure 2B, the PKS includes several units, such as the starter acyl transferase (SAT) domain selects an acetyl-CoA starter unit, and the ketoacyl synthase (KS)-acyl transferase (AT)-acyl carrier protein (ACP) domains extend this starter unit five times with malonyl-CoA in five successive decarboxylative Claisen condensation cycles. The methyltransferase (MT) domain conducts a single C-methylation at C-4 carbon. The reactive hexaketide chain then undergoes a product template (PT) domain-mediated C-2 to C-7 aldol cyclization to afford the first aromatic ring chemical **3** (Li et al., 2010; Xu et al., 2013; Liu et al., 2015). The first ring chemical **3** follows reductive release chemical **4** catalyzed by reductive releasedomain (R) (Bailey et al., 2007). Mrpig C catalyzes the transformation of chemical **4** to **5** via ω-1 carbonyl to the alcohol. The chemical **5** has been isolated and identified from the broth of *Monascus ruber* mutant (Liu et al., 2016).

Mrpig N is critical to morphing the chemical **5** into the bicyclic pyran-containing azaphilone core, which is confirmed by *in vivo* experiment via knockout of Mrpig N (Chen et al., 2017). Hydroxylation at C-4 carbon of chemical **5** is catalyzed by a FAD-dependent monooxygenase encoded by Mrpig N, which leads to de-aromatization of the ring and then induce keto-enol tautomerization at the C-1 aldehyde. Finally, the condensation between the C-1 enol and the C-9 carbonyl to afford the pyran ring, i.e., the key azaphilone chemical **6**. Chemical **7** (Woo et al., 2014) and chemical **8**/**9** (Jongrungruangchok et al., 2004) are also isolated and identified, which are resulted from the further modification of the key azaphilone chemical **6** by enzymatic or non-enzymatic reaction. Fatty acid 3-oxo-octanoic acid (**10**) and 3-oxo-decanoic acid (**11**) are produced catalyzed by FAS (Balakrishnan et al., 2014c; Liang et al., 2018). Mrpig D encodes acyltransferase, which catalyzes the transferring of the fatty acids (**10**/**11**) to the C-4 alcohol of chemical **7** and formation of the anticipated intermediate **12**.

### Formation of the Tricyclic Ring of *Monascus* Pigments

Different from most of azaphilones, the tricyclic ring of *Monascus* pigments is established by intramolecular Knoevenagel aldol condensations of key intermediate **12**. Mrpig E, an ortholog of mpp C (98% identity), determines the region-selectivity of the spontaneous Knoevenagel condensation. It is proposed that the NAD(P)H-dependent oxidoreductase encoded by Mrpig E gene catalyzes the reduction of compound **12** into a chemical **13**. This reductive reaction eliminates the π-conjugated system in chemical **12** and makes C-5 carbon of chemical **13** a better electron acceptor. Thus, the anticipated linear tricyclic intermediate **14** is produced by Knoevenagel condensation. A Δmpp C mutant produces monasfluore A/B (**15**/**16**) by Knoevenagel condensation between α carbon in the 3-oxo-fatty acyl moiety and the C-3 carbonyl group of chemical **12** (Balakrishnan et al., 2014b). In general, majority of chemicals **12** are channeled by Mpig E through the main pathway toward the linear tricyclic intermediate **14** and the angular chemicals **15/16** are also yielded at a low level (Huang et al., 2008).

An oxidase, encoded by mpp G gene (corresponding to Mrpig F in Figure 2A), controls transformation of intermediate **14** into orange *Monascus* pigments (**1**/**2**). A Δmmp G mutant abolishes the biosynthesis of orange *Monascus* pigments while no significant alteration the level of yellow *Monascus* pigments **(17/18)**. This result indicates that yellow *Monascus* pigments can be biosynthesized independently from orange *Monascus* pigments. On the other hand, feeding of yellow *Monascus* pigment (**17**) to ΔMpPKS5 mutant also excludes the possibility of conversion of yellow *Monascus* pigment into the corresponding orange one (**1**) (Balakrishnan et al., 2017b). Similarly, a reductase, encoded by mpp E gene (corresponding to Mrpig H in Figure 2A), controls the conversion of the intermediate **14** into yellow *Monascus* pigments (**17/18**) (Balakrishnan et al., 2017a). The key role of intermediate **14** to the biosynthesis of *Monascus* pigments is also confirmed via analysis of Mrpig E by gene disruption, complementation and overexpression in *Monascus ruber* (Liu et al., 2014). The results demonstrate that the Mrpig E deletion strain failes to produce orange *Monascus* pigments and the Mrpig E complementation strain recovers the ability to production of orange *Monascus* pigments. However, there are still detectable yellow *Monascus* pigments in the broth after culture of Δmpp E mutant (Balakrishnan et al., 2017a). Further experiment indicates the Δmpp DEG (mpp D corresponding to Mrpig G in Figure 2A) mutant remains the ability for production of orange *Monascus* pigments as well as yellow ones (Balakrishnan et al., 2017a). These results hint that multiple discrete routes may be involved in the biosynthesis of *Monascus* pigments.

### Production of Extracellular Orange *Monascus* Pigments

**FIGURE 3:**
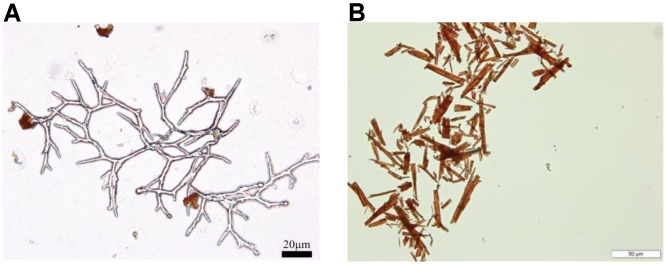
Production of extracellular orange *Monascus* pigments. **(A)** Mycelia morphology of submerged culture (bar = 20 μm); **(B)** needle-like crystals of orange *Monascus* pigments (bar = 50 μm).

As shown in Figure 2A, there is Mrpig P gene in the gene clusters for biosynthesis of *Monascu*s pigments. The Mrpig P gene, which encodes an efflux transporter, is also observed in other filamentous fungi, such as *Aspergillus niger* (Zabala et al., 2012). This fact indicates that there is possibility of the efflux orange *Monascus* pigments into its extracellular broth during submerged culture. In fact, there is report that a hyper-pigment-producing mutant, derived from *Monascus* Kaoliang F-2 through a series of mutagenesis steps, produces pigments existing as lumps together with some viscous substances outside the cells (Lin and Lizuka, 1982). Recently, a collection of crystal *Monascus* pigments by further purification is reported in literature (Vendruscolo et al., 2014) and production of extracellular *Monascus* pigments is also confirmed (Lu et al., 2018). The mycelia morphology of submerged culture is observed by optical microscope. There are many lumps of pigments sticking on the mycelia surfaces (Figure 3A). Those pigments are collected by membrane filtration, which is confirmed majorly as orange *Monascus* pigments (**1/2**) (Figure 3B) and a few of yellow *Monascus* pigments (**17/18**). This fact eliminates the long time mistaken concept that orange (yellow) *Monascus* pigments are predominantly cell-bound, including both intracellular and surface-bound pigments (Lin and Lizuka, 1982; Chen and Johns, 1994; Mapari et al., 2012). Some even manage to export the intracellular or cell-bound *Monascus* pigments during submerged culture by addition of non-ionic surfactant (Hu et al., 2012) or the antifungal agent fluconazole (Kolia et al., 2017). This mistake may be attributed to the confusion between water-insoluble *Monascus* pigments and intracellular ones.

## Production of *Monascus*-Like Pigments

Filamentous fungi are large-scale producers of secondary metabolite with azaphilone structure. Orange *Monascus* pigments are a kind of secondary metabolites with azaphilone structure. Some *Talaromyces* species (Woo et al., 2014) or *Penicillium* species (Wei et al., 2017) also produce pigments with similar structure of *Monascus* pigments.

**FIGURE 4:**
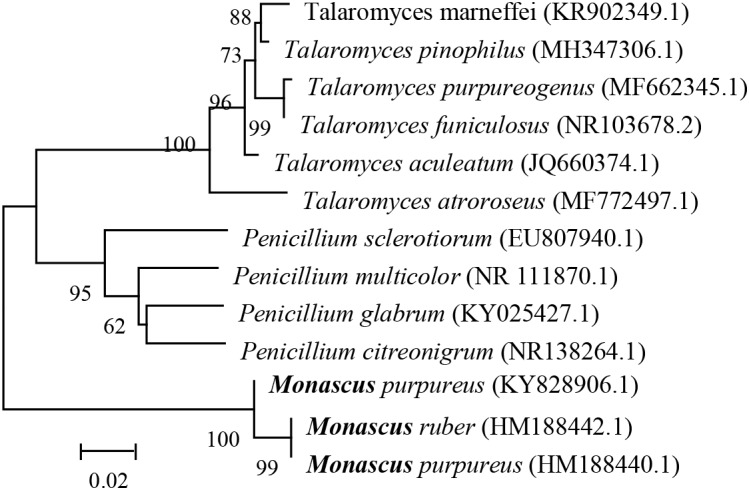
Multigene phylogenies of some filamentous fungi.

Internal transcribed spacer (ITS) sequence of *Penicillium* species, *Talaromyces* species, and *Monascus* species, were obtained from NCBI. Data set were aligned using MEGA5 software. The ITS sequences of thirteen strains were selected and aligned by ClustalW. Then, the unaligned parts at both ends were deleted. Finally, the aligned sequences were used for phylogenetic analysis. A phylogenetic tree with the ITS region of *P. sclerotiorum* FS50 was constructed by Neighbor-Joining method with 1000 bootstrap replication using MEGA5 software based on the distance matrix for all pairwise sequence combinations (Figure 4). *Talaromyces* species were highly homologous to *Penicillium* species. The homology between *Penicillium* species and *Monascus* species was more closed than that of *Talaromyces* species and *Monascus* species.

**FIGURE 5:**
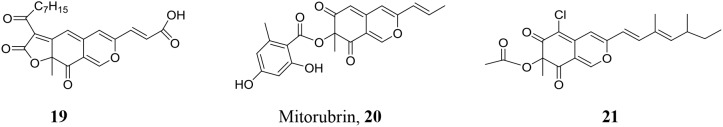
*Monascus*-like pigments produced by different filamentous fungi.

Just like secondary metabolites of *Monascus* species, *T. marneffei* (formerly known as *P*. *marneffei*) produces both *Monascus* pigments and citrinin (Woo et al., 2014). It indicates that orange *Monascus* pigments (**1**/**2**) can be produced by non-*Monascus* sp. However, *T. marneffei* is the most important thermal dimorphic fungus causing respiratory, skin and systemic mycosis (Woo et al., 2007), which is excluded from food microbiology for production of *Monascus* pigments. *P. purpurogenum* (formerly known as *P*. *purpurogenum*) produces *Monascus* pigment homolog (**19** of Figure 5) besides the conventional *Monascus* pigments (Arai et al., 2015). *T. purpurogenus* is recognized to be interesting industrially (Mapari et al., 2012), but mycotoxins, such as rubratoxin A and B and luteoskyrin, are also produced (Frisvad et al., 2013). *T*. *atroroseus* produces mitorubrin (**20**) along with orange *Monascus* pigments without being accompanied by mycotoxin synthesis (Frisvad et al., 2013). Mitorubrin is a azaphilone chemical, which has similar structure to orange *Monascus* pigments except lack of lactone ring structure. Then, *T*. *atroroseus* is regarded as potential strain for replacement of *Monascus* sp. to produce pigments (Mapari et al., 2009). Those results indicate that *Monascus* pigment homologs (**19**) or *Monascus*-like pigments (**20**), even *Monascus* pigments, can be produced by *Talaromyces* species.

In spite of the close relationship between *Penicillium* species and *Monascus* species, there is no report on production of *Monascus* pigments by *Penicillium* species. However, *Penicillium* species can be utilized for production of *Monascus*-like azaphilones. In 1940, sclerotiorin (**21**) was isolated and identified from the fermentation broth of *P*. *sclerotiorum* (Curtin, 1940). This is a *Monascus*-like pigment with the replacement of C-6 hydrogen with chlorine. Production of sclerotiorin as well as its derivates by other *Penicillium* species is also reported (Hemtasin et al., 2016). Those *Penicillium* species do not co-produce citrinin or any other known mycotoxins and non-pathogenic to humans, which are potential strains for production of food pigments (Mapari et al., 2008; Dufossé et al., 2014; Gomes and Takahashi, 2016).

**FIGURE 6:**
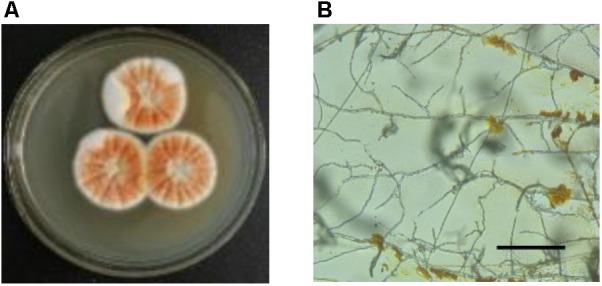
Accumulation of sclerotiorin with different culture modes. **(A)** Mycelia colony of solid-state fermentation on plate; **(B)** extracellular pigments sticking on aerial hyphae (bar = 100 μm). For detailed microbial culture and experimental procedure, please reference (Xin et al., 2018).

Traditionally, *Monascus* pigments are produced by solid-state fermentation on rice. In 1975, submerged culture was introduced into the production of *Monascus* pigments (Yoshimura et al., 1975). During submerged culture, mycelia of *Monascus* sp. acts as planktonic cells and extracellular *Monascus* pigments are produced (Figure 3A). However, sclerotiorin is usually produced by solid-sate fermentation (Lin et al., 2015; Wei et al., 2017) or submerged culture without stirring (mycelia of *Penicillium* sp. acting as mycelial mat on air-liquid surface) (Curtin, 1940; Gomes and Takahashi, 2016). There are few reports on production of sclerotiorin by submerged culture with stirring (Celestino et al., 2014). Marine-derived strain *P. sclerotiorum* FS-50 (GenBank accession number EU 807940) was isolated from the sediment of South China Sea and stored in Guangdong Microbial Culture Collection Center (GDMCC) (Lin et al., 2015). Microbial fermentation in Czapek medium (agar 15g, glucose 20 g, NaNO_3_ 3 g, KH_2_PO_4_ 1 g, KCl 0.5 g, MgSO_4_⋅7H_2_O 0.5 g, FeSO_4_⋅7H_2_O 0.1 g, CuSO_4_⋅5H_2_O 0.05 g, per liter of tap water with natural pH) was carried out in different modes for 7 days. In solid-state plate culture, yellow color was observed on the mycelial surfaces (Figure 6A). Further microscopic observation indicated that the yellow pigment was attached on aerial hyphae (Figure 6B). The production of extracellular pigments is very similar to that of orange *Monascus* pigments (Figure 3B). The extracellular yellow pigment was identified as sclerotiorin. Very interestingly, submerged culture in the Czapek medium in the absence of agar with stirring and without stirring exhibited very different characters. Mycelial mat formed on the air-liquid surface during submerged culture without stirring and the mycelial mat exhibited yellowish due to containing sclerotiorin. On the contrary, mycelia exhibited as planktonic cells during submerged culture with stirring and there was nearly no sclerotiorin accumulation. The mechanism behind the phenomenon remains blurry (Xin et al., 2018).

## Chemical Modification of *Monascus* Pigments

Orange *Monascus* pigments (**1**, **2**) as well as *Monascus*-like pigments (**19**, **20**, **21**) are the secondary metabolites of microbial fermentation. The chemical diversity is limited by the screened microbial strains. Fortunately, this subclass of azaphilones can be further modified chemically.

### Replacement of Pyranyl Oxygen With Primary Amine

**FIGURE 7:**
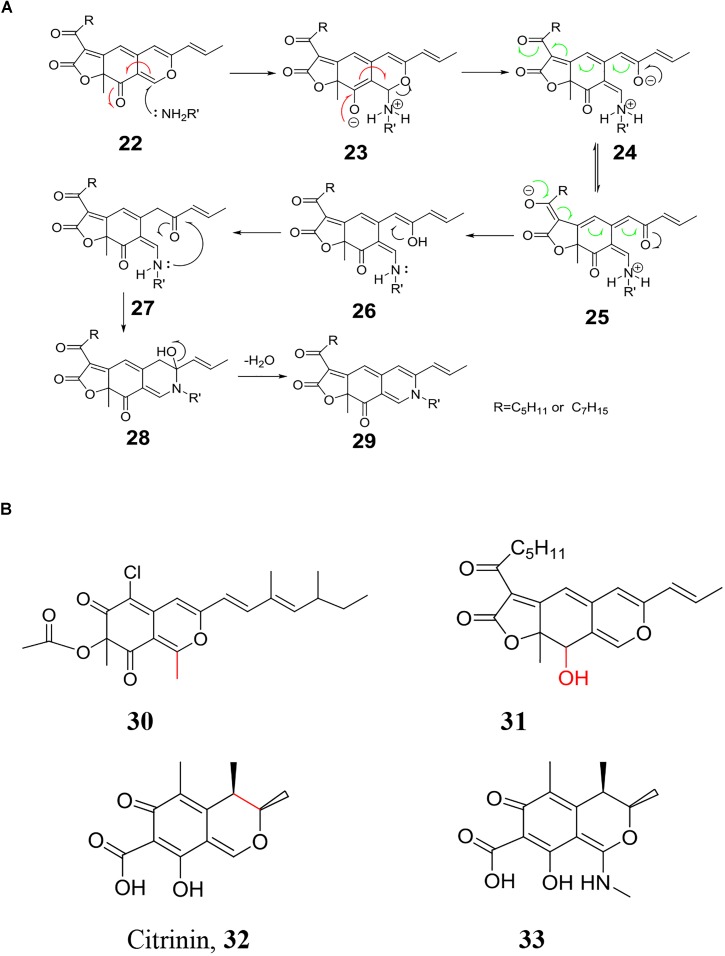
Chemical modification of orange *Monascus* pigments with primary amine. **(A)** Replacement of pyranyl oxygen in orange *Monascus* pigments with primary amine; **(B)** key structure to replacement of oxygen with primary amine.

Orange *Monascus* pigments (Figure 1) as well as *Monascus*-like pigments are a subclass of azaphilones with the characteristic reaction, i.e., replacement of pyranyl oxygen with primary amine form red pigments (Figure 7A). This characteristic reaction is known as aminophilic reaction in the following text. Aminophilic reaction begins with Michael addition of a nucleophilic primary amine to the electrophilic C-1 carbon and results in the formation of carbinolamine (**22**), in which involves electric transfer in the conjugated double bond system (red arrows). Chemical **22** undergoes C-O bond cleavage to generate enamines (**23**, **24**), which involves electric transfer in the other conjugated double bond system (green arrows). An intramolecular proton transfers from nitrogen to oxygen gives enamine (**25**) followed by **26**. Nucleophilic attack on the C-2 carbonyl by the lone electron pair of enamines (**27**) results in the formation of **28**, which undergoes dehydration to give the nitrogen-containing orange *Monascus* pigment derivates (OMPDs) with various primary amine residues (**29**) (Wei and Yao, 2005). In this possible mechanism, azaphilone structure of C-1 hydrogen, two sets of conjugated double bond systems (green arrows and red arrows) are necessary for the aminophilic reaction.

The mechanism of aminophilic reaction is confirmed by the reactive activity of some azaphilones (Figure 7B). Similar to orange *Monascus* pigments, replacement of pyranyl oxygen in sclerotiorin (**21**) with primary amine occurs smoothly while methylation of C-1 carbon in sclerotiorin (**30**) blocks this chemical reaction (Gomes and Takahashi, 2016). Hydroxylation of C-3 carbon (**31**) (Zheng et al., 2010) or cyclization of C-3 carbon to formation of *Monascus* metabolites with strong blue fluorescence (**15, 16**) (Huang et al., 2008) also prevents this chemical reaction to occur due to the lack of conjugated double bond system (red arrows in Figure 7A). With the same principle, replacement of pyranyl oxygen in yellow *Monascus* pigments (**17, 18**) with primary amine is also blocked due to the lack of conjugated double bond system (green arrows in Figure 7A). There is no chemical reaction between yellow *Monascus* pigments (**17, 18**) and primary amine, which has been applied for separation of yellow *Monascus* pigments from orange ones after microbial fermentation (Zhong et al., 2015). Similarly, primary amine also fails to replacement of oxygen in citrinin (**32**). However, it is also reported that methyl amine attacks the C-1 carbon to formation of new compound **33** (Natsume et al., 1988).

### Orange *Monascus* Pigment Derivates

**FIGURE 8:**
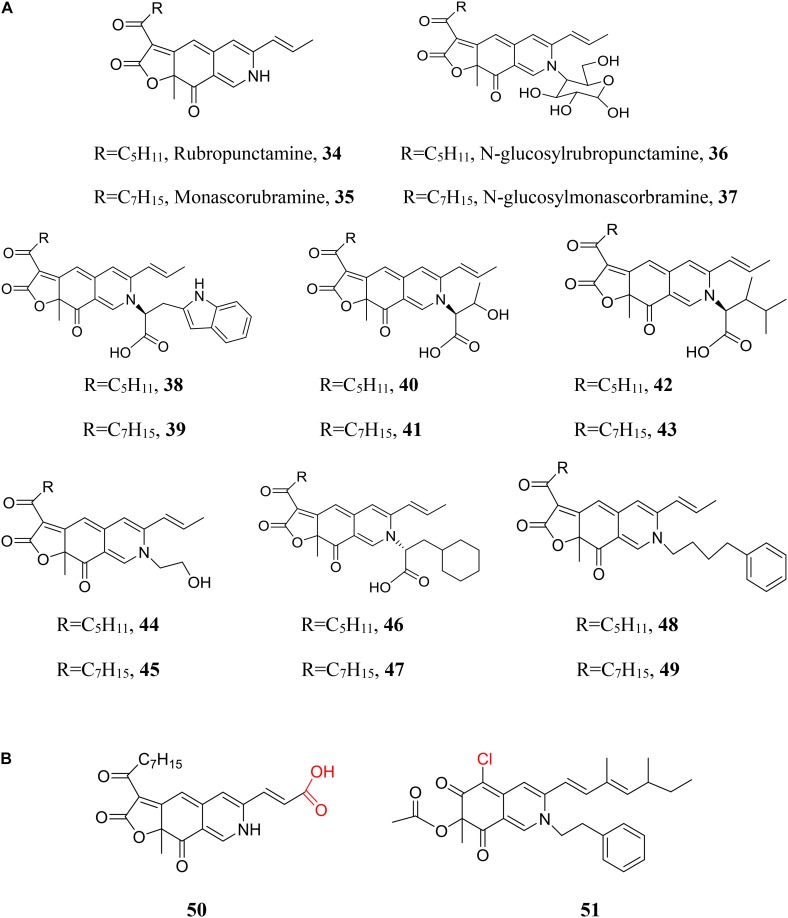
Chemical modification of azaphilones with primary amines. **(A)** Diversification of *Monascus* pigments with various primary amines; **(B)** diversification of *Monascus* pigment homologs or *Monascus*-like pigments with primary amine.

Aminophilic reaction of orange *Monascus* pigments provides a chance to diversification of *Monascus* pigments due to the large number of primary amines. The well-known red *Monascus* pigments, rubropunctamine (**34**) and monascorubramine (**35**), are a pair of OMPDs with ammonia residue (Figure 8A). The primary amines of OMPDs (**29**) may be natural or non-natural primary amines. Many OMPDs with various natural primary amines, such as glucosamine (**36**, **37**) (Hajjaj et al., 1997), *L*-amino acids, such as *L*-tryptophan (**38**, **39**) (Kim et al., 2007a), *L*- threonine (**40**, **41)** (Jeun et al., 2008), and *L*-leucine (**42**, **43**) (Sun et al., 2012), ethanol amine (**44**, **45**) (Frisvad et al., 2013), even non-natural *D*-amino acid (**46**, **47**) (Kim et al., 2007b), are isolated from fermentation broth. At the same time, OMPDs with various non-natural primary amines, such as 4-phenylbutylamine (**48**, **49**) (Choe et al., 2012), are also achieved by direct chemical modification of orange *Monascus* pigments.

Compared to enlarging the chemical library by various primary amines (Figure 8A), replacement of orange *Monascus* pigments with *Monascus* pigment homologs or *Monascus*-like pigments may be a more efficient strategy (Figure 8B). Corresponding to *Monascus* pigment homolog (**19**), derivate with ammonia residue (**50**) is also isolated (Ogihara et al., 2000). A series of sclerotiorin derivates with different non-natural primary amines are also prepared by chemical reaction between sclerotiorin and primary amines (Wei et al., 2017). Especially, a sclerotiorin derivate with phenylethylamine residue (**51**) had been produced by sequential fungal fermentation-biotransformation process (Gomes and Takahashi, 2016).

Preparation of OMPDs (**29**) involves biosynthesis of orange *Monascus* pigments and chemical modification of orange *Monascus* pigments with primary amine. One way is separation of orange *Monascus* pigments after *Monascus* fermentation and then carrying out chemical reaction between orange *Monascus* pigments with natural or non-natural primary amine. Method for solvent extraction of orange *Monascus* pigments from fermentation broth as well as the purification of orange *Monascus* pigments from the extractant by silica gel adsorption is suggested (Choe et al., 2012). The chemical reaction involves the water-insoluble orange *Monascus* pigments and water-soluble primary amines. An ethanol aqueous solution (Lin et al., 1992), the organic solvent triethylamine (Wei et al., 2017), a non-ionic surfactant micelle aqueous solution (Xiong et al., 2015), even an aqueous solution with adsorbent (Achard et al., 2012) are utilized as reaction medium to enhance the heterogeneous reaction rate. Alternatively, OMPDs can also be produced directly by microbial fermentation. Red Yeast Rice is a traditional Chinese food colorants produced directly by solid-state fermentation of *Monascus* species on rice, in which red pigments (i.e., a mixture with many kind of OMPDs) are produced due to the presence of a large number of amino acids or peptides in rice. However, whether involving enzymatic catalysis in the heterogeneous chemical reaction during the fermentation process remains unclear. Based on this fact, addition of a relatively excess of natural primary amines, such as amino acids, into the fermentation medium is applied for direct production of OMPDs (Blanc et al., 1994; Jung et al., 2003; Woo et al., 2014) and the OMPDs are further separated and purified by silica column chromatography (Jung et al., 2003). The chemical constituents of OMPDs are complicated due to the microbial metabolism involving complex primary amines, which makes silica gel column chromatography only suitable for preparation of a small amount of sample.

### Characters of *Monascus* Pigment Derivates

Orange *Monascus* pigments are water-insoluble pigments with characteristic absorbance wavelength at approximately 470 nm in an ethanol aqueous solution. The low water-solubility limits the application of orange *Monascus* pigments as food colorant. Even red *Monascus* pigments (**34**, **35**) remains a limited solubility in water and only solid state of Red Yeast Rice is utilized traditionally as food colorant. It is reported that replacement of ammonia with various amino acid residues strongly alters the hydrophobicity of OMPDs. At the same time, the characteristic absorbance wavelength also transfers from red *Monascus* pigments (**34**, **35**) at approximately 508 nm to varying from 498 to 525 nm depending on a special amino acid (Jung et al., 2003). These characters are further confirmed by examination of the solubility of OMPDs with water-soluble amino acids (Wong and Koehler, 1983). Furthermore, the stability of OMPDs is also influenced by the primary amine structure. It is well known that photo-instability is the drawback of *Monascus* pigments (Sweeny et al., 1981). The photo-stability of OMPDs with various amino acid residues is enhanced markedly under sunlight irradiation condition compared to the red *Monascus* pigments (**34**, **35**). More interestingly, OMPDs with amino acid residues exhibit stabile at nearly neutral pH while red *Monascus* pigments (**34**, **35**) are relatively stable under acidic condition (Jung et al., 2005, 2011). At the same time, thermal stability of OMPDs is enhanced compared to orange *Monascus* pigments themselves (Vendruscolo et al., 2013). Red pigments produced by *P. purpurogenum* GH2 also shows a relatively higher thermal stability (Morales-Oyervides et al., 2015). The characters of red color, water-soluble, and high stability at neutral pH makes OMPDs with amino acid residues potential of food colorant. The food additives standard of a red colorant has already been issued and updated several times by government of the People Republic of China (Red *Monascus* Pigments^®^, GB1886.181-2016). The major constituents of Red *Monascus* Pigments^®^ are OMPDs with various amino acid/peptide residues.

**FIGURE 9:**
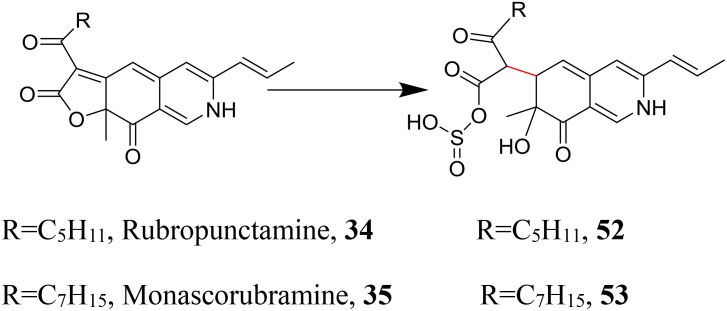
Further chemical modification of red *Monascus* pigments.

Native yellow *Monascus* pigments (**17**, **18**) are hydrophobic/water-insoluble with its characteristic absorbance wavelength approximately 400 nm. Very luckily, Red *Monascus* pigments^®^ can be further chemically modified with sodium hydrosulfite to produce water-soluble yellow pigments. Novel compounds (**52**, **53**) are isolated and identified from the water-soluble yellow *Monascus* pigments, which exhibits characteristic absorbance wavelength at 468 nm. Chemical transformation of red *Monascus* pigments (**34**, **35**) into compounds (**52**, **53**) by reduction and sulfonation reaction (Figure 9) may be involved in the chemical modification of Red *Monascus* Pigments^®^ with sodium hydrosulfite (Yang et al., 2018). The safety of this water-soluble yellow *Monascus* pigments is also evaluated and the corresponding food additive standard has been issued recently by the Chinese government (Yellow *Monascus* Pigments^®^, GB 1886-66-2015).

## *Monascus* Pigments Acting as Enzyme Inhibitor

Red Yeast Rice as a traditional Chinese medicine involves many bioactive constituents (Ma et al., 2000). At the same time, *Monascus* pigments are series of azaphilones. Azaphilones are a class of fungal metabolites with diverse bioactivities (Osmanova et al., 2010). There are many reports about the bioactivity of *Monascus* pigments, such as the antimicrobial activities of OMPDs with amino acid residues (Kim et al., 2006) and the anti-cancer activity of *Monascus* pigments (Zheng et al., 2010; Yang et al., 2014), as well as *Monascus*-like pigments, such as the antifouling activity of the sclerotiorin derivatives (Wei et al., 2017). We restricted the scope within pigment bioactivity acting as enzyme inhibitors in the present work.

### Pancreatic Lipase Inhibitor and Obesity

Physical inactivity and overeating are the main causes of obesity. Hence, controlling the digestion of dietary lipids is a promising approach to treat obesity. Lipstatin is a β-lactone molecule which controls the digestive activity of pancreatic lipases and thus controls the fat absorption in the small intestine, which is utilized as anti-obesity medicine (Kumar and Dubey, 2015). On the other hand, differentiation and hypertrophy of adipocytes are fundamental processes of obesity. The differentiation of pre-adipocytes into adipocytes involves exposure of a confluent, quiescent population of cells to a variety of effectors that activate a cascade of transcription factors. This cascade begins with the CCAAT/enhancer-binding protein (C/EBP) β and C/EBP δ, which finally induce the expression of C/EBP α and peroxisome proliferator activated receptor (PPAR) γ. These transcription factors coordinate the expression of genes involved in creating and maintaining the adipocyte phenotype (Rosen et al., 2000).

Orange *Monascus* pigment derivates with different amino acid residues, such as unnatural amino acids (Kim et al., 2007b), L/D-amino acids (Kim et al., 2007a), are screened for pancreatic lipase inhibitor. OMPD with *L*-tryptophan residue (**38**, **39**) exhibits the highest inhibitory activity with IC_50_ value 61.2 mM (Kim et al., 2007a). Administration of the jelly food with OMPDs (**38**, **39**) also confirms that the total cholesterol, LDL (low-density lipoprotein) cholesterol and triacylglycerol levels in the mouse serum are lowered. Significantly reduction of subcutaneous fat and visceral fat amounts in mice are also observed using micro CT images of mice tissues (Nam et al., 2014). In addition, experiments also found that OMPDs with 4-phenylbutylamine (PBA, **48**/**49**) or 2-(*p*-toyly) ethylamine (TEA) residue also shows an inhibitory activity against adipogenic differentiation in 3T3-L1 cells. The transcription factors PPAR γ and C/EBP α are down-regulated by the PBA derivative and the TEA derivative at 10 μM. Both the number and droplet size of fatty cells are reduced by treatment with the inhibitory derivatives (Choe et al., 2012).

Besides OMPDs exhibiting as anti-obesity agents, yellow *Monascus* pigments, ankaflavin (**17**) and monascin (**18**), exhibit anti-obesity effect via the suppression of differentiation and lipogenesis (Lee et al., 2013). Red Yeast Rice with high content of yellow *Monascus* pigments, market known as Ankascin^®^, has already been commercialized and approved by the US FDA as a new dietary ingredient (NDI). The anti-obesity effect of yellow *Monascus* pigments is related to down-regulate the transcription factors C/EBP β/PPAR γ expression, inhibit lipogenesis by increasing lipase activity, and suppress Niemann-Pick C1 Like 1 (NPC1L1) protein expression associated with small intestine tissue lipid absorption (Jou et al., 2010; Lee et al., 2013). The effect of yellow *Monascus* pigments on obesity-related-diseases is also investigated, such as hyperlipidemia, i.e., down-regulation of total cholesterol, triglyceride, LDC cholesterol level in the serum, and up-regulation of low-density lipoprotein (HDL) cholesterol level in serum (Lee et al., 2010), steatohepatitis (Hsu et al., 2014), and hyperglycemia (Hsu et al., 2013).

### HMG-CoA Reductase Inhibitor and Hyperlipidemia

Anabolism of cholesterol occurs by transfer of acetyl-CoA from the mitochondrion to the cytosol. Acetyl-CoA can be sequentially converted to hydroxy-methyl-glutaryl coenzyme (HMG-CoA), mevalonate, squalene, lanosterol, and cholesterol. The conversion reaction of HMG-CoA to mevalonate, which is catalyzed by HMG-CoA reductase, is known to be a key rate-limiting step in cholesterol biosynthesis. Activity regulation of HMG-CoA reductase can control the cholesterol content in the body and then hypercholesterolemia. Lovastatin, produced by fermentation of *Aspergillus terreus* (Boruta and Bizukojc, 2017) as well as *Monascus* sp. (Lu et al., 2013), is a well-known inhibitor of HMG-CoA reductase and widely used as a hypercholesterolemia drug for reduction of plasma cholesterol levels in humans.

Orange *Monascus* pigment derivates with different amino acid residues are further screened for HMG-CoA reductase inhibitor (Jeun et al., 2008). Using orange *Monascus* pigments (**1**, **2**) as control (exhibiting high inhibitor activity to HMG-CoA reductase), experimental result indicates that OMPDs with threonine residue (**40**, **41**) have good exhibitory activity (38%) while derivates with other amino acid residues shows low activity compared to orange *Monascus* pigments. *In vivo* tests using female C57BL/6 mice further confirms the total cholesterol level of mouse serum is reduced by 8–9% with OMPDs (**40**, **41**) and by 16% with orange *Monascus* pigments. Supplementation with OMPDs (**40**, **41**) and orange *Monascus* pigment decreases the LDL cholesterol level by 18–26% and increases the HDL cholesterol level by 1–9%.

Cellular lipid and cholesterol metabolism play either a direct or indirect role in membrane integrity. In particular, cholesterol is proposed as an integral part of lipid raft structure (Garcia-Fernandez et al., 2017). The replication of hepatitis C virus (HCV) depends on the host cells to provide cholesterol as raw materials. A block of mevalonate biosynthesis pathway in host cells should inhibit HCV replication. Orange *Monascus* pigments as well as their amino acid derivates are further screened for HCV antiviral agent. A group of OMPDs with various amino acid residues, such as leucine (**42**, **43**), significantly inhibit HCV replication (Sun et al., 2012). Similarly, lipid rafts in cell membrane of pathogen methicillin resistant *Staphylococcus aureus* (MRSA) is also related to its multidrug-resistant. Interfering with cholesterol biosynthesis pathway is also potential for dealing with multidrug-resistant of MRSA (Garcia-Fernandez et al., 2017).

### PTP1B Inhibitor and Hyperglycemia

Anti-diabetic thiazolidinedione (TZD) drugs, such as rosiglitazone and pioglitazone, are PPAR-γ agonists and used to manage obesity-related insulin resistance and type 2 diabetes (hypoglycemic agent). Alternatively, protein-tyrosine phosphatases (PTP) have an important role in the regulation of insulin signal transduction. PTP1B, protein tyrosine phosphatase 1B, is a prototype non-receptor cytoplasmic PTP enzyme that negatively regulates insulin and leptin signaling pathways (Ahmad et al., 1997). Thus, PTP1B inhibitor is a potential agent for the treatment of diabetes.

An interesting paper, using a new method of ultrafiltration-based LC-MS to directly screening of PTP1B inhibitor from the traditional Red Yeast Rice, has been published recently. The experimental result shows that monascorubramine (**35**) possesses inhibitory activity toward PTP1B and the anti-diabetic effect of Chinese Red Yeast Rice is partially attributed to potential PTP1B inhibitory activity of monascorubramine (Jin et al., 2016). Monascorubramine acting as PTP1B inhibitor is based on the chemical library of native Red Yeast Rice. It reasonably deduces that there is more efficient PTP1B inhibitor in the library of bio-and chemo-diversification of native *Monascus* pigments as well as *Monascus*-like pigments (Figure 8).

## Summary

Traditionally, *Monascus* pigments are produced natively by solid-state microbial fermentation on rice. The Red Yeast Rice has been utilized as food colorant and traditional Chinese medicine for more than 1000 years (Ma et al., 2000; Chen et al., 2015). The safety of Red Yeast Rice has been confirmed by Chinese as well as other East Asia people. Due to the complex secondary metabolites, such as citrinin (**32**), Red Yeast Rice is still be excluded in the list of food additives by the European Food Safety Authority (EFSA) (Kallscheuer, 2018).

With the progress on study of the biosynthetic pathway of *Monascus* pigments, it is recognized that red pigments of *Monascus* fermentation result from the chemical modification of orange *Monascus* pigments with different primary amines. By application of submerged culture other than traditional solid-state fermentation technique, Red *Monascus* Pigments^®^, the major constitutes are OMPDs with various amino acid/peptide residues, can be produced commercially. In the updated food additive standard of Chinese government (GB1886.181-2016), the content of citrinin is also strictly restrained. The utilization of red colorant from filamentous fungi in food industry is the reality (Dufosse, 2018). With the further progress on chemical modification of orange *Monascus* pigments, production of OMPDs (**29**) with a single primary amine residue becomes possible. The red colorants from filamentous fungi acting as food additives will be finally approved by more and more countries.

Many bioactive components have been isolated and identified from the Chinese traditional medicine Red Yeast Rice. Yellow *Monascus* pigments exhibit anti-obesity activity as well as obesity-related-diseases, such as hyperlipidemia, steatohepatitis, and hyperglycemia (Hsu and Pan, 2014). Red Yeast Rice with high content of yellow *Monascus* pigments, market known as Ankascin^®^, has been approved by the US FDA as a new dietary ingredient (NDI). With the diversification of the chemical structures of orange *Monascus* pigments/*Monascus*-like pigments (Figure 8), a very large chemical library should provide more chances for screening bioactive compounds. Besides yellow *Monascus* pigments, it is foreseeable that more and more OMPDs (**29**) will be added into the list of functional food.

## Author Contributions

LL wrote the section of “Introduction”, “Biosynthesis of *Monascus* Pigments”, and “*Monascus* Pigments Acting as Enzyme Inhibitor”. YH wrote the section of “Chemical Modification of *Monascus* Pigments”. QX wrote the section of “Production of *Monascus*-Like Pigments”. JZ and ZW read the whole manuscript.

## Conflict of Interest Statement

The authors declare that the research was conducted in the absence of any commercial or financial relationships that could be construed as a potential conflict of interest.
